# Non-linear Response to Cell Number Revealed and Eliminated From Long-Term Tracheid Measurements of Scots Pine in Southern Siberia

**DOI:** 10.3389/fpls.2021.719796

**Published:** 2021-10-04

**Authors:** Elena A. Babushkina, Dmitry R. Dergunov, Liliana V. Belokopytova, Dina F. Zhirnova, Keshav K. Upadhyay, Shri K. Tripathi, Mikhail S. Zharkov, Eugene A. Vaganov

**Affiliations:** ^1^Khakass Technical Institute, Siberian Federal University, Abakan, Russia; ^2^Siberian Federal University, Krasnoyarsk, Russia; ^3^Department of Forestry, Mizoram University, Aizwal, India; ^4^Sukachev Institute of Forest, Siberian Branch of the Russian Academy of Science, Krasnoyarsk, Russia

**Keywords:** dendroclimatology, quantitative wood anatomy (QWA), cell number, tracheid radial diameter, cell wall thickness, *Pinus sylvestris* (L), legacy effect

## Abstract

Dendroclimatic research offers insight into tree growth–climate response as a solution to the forward problem and provides reconstructions of climatic variables as products of the reverse problem. Methodological developments in dendroclimatology have led to the inclusion of a variety of tree growth parameters in this field. Tree-ring traits developed during short time intervals of a growing season can potentially provide a finer temporal scale of both dendroclimatic applications and offer a better understanding of the mechanisms of tree growth reaction to climatic variations. Furthermore, the transition from classical dendroclimatic studies based on a single integral variable (tree-ring width) to the modern multitude of quantitative variables (e.g., wood anatomical structure) adds a lot of complexity, which mainly arises from intrinsic feedbacks between wood traits and muddles seasonality of registered climatic signal. This study utilized life-long wood anatomical measurements of 150- to 280-year-old trees of *Pinus sylvestris* L. growing in a moisture-sensitive habitat of the forest-steppe of Southern Siberia (Russia) to investigate and eliminate legacy effect from cell production in tracheid traits. Anatomical parameters were calculated to describe the results of the three main subsequent stages of conifer xylem tracheid development, namely, cell number per radial file in the ring, mean and maximum cell radial diameter, and mean and maximum cell-wall thickness. Although tree-ring width was almost directly proportional to cell number, non-linear relationships with cell number were revealed in tracheid measurements. They exhibited a stronger relationship in the areas of narrow rings and stable anatomical structure in wider rings. The exponential models proposed in this study demonstrated these relationships in numerical terms with morphometric meaning. The ratio of anatomical measurements to their modeled values was used to develop long-term anatomical chronologies, which proved to retain information about climatic fluctuations independent of tree-ring width (cell number), despite decreased common signal.

## Introduction

Climate has a profound effect on the growth and distributional range of tree species, and therefore, improved understanding of the tree growth–climate relationship will be helpful in their better management and conservation under changing climate scenarios (Weiskopf et al., 2020; Upadhyay et al., 2021). This becomes imperative, especially in areas like South Siberia, where forests growing alongside arid forest lines are vulnerable to even a small change in moisture regime (Xu et al., 2017). Dendroclimatic studies enable us to provide crucial empirical data on the long-term effect of climate on forest tree growth and development. Classical dendroclimatology determines the climatic factors responsible for significant variations in quantitative characteristics of tree rings and provides their tree-ring-based reconstruction under reverse task (Fritts, 1976; Hughes et al., 2010; Schweingruber, 2012). Such studies were initially started with a basic feature, tree-ring width, as a recorder of climate effect on tree growth (Kapteyn, 1914; Douglass, 1920, 1925; Bailey, 1948; Glock, 1955), and progressed with the spectrum of variables, namely, the width of early- and latewood zones, wood density characteristics, isotopic and chemical composition, and finally, quantitative wood anatomy (QWA) down to the scale of cells (Fry and Chalk, 1957; Zahner et al., 1964; Hughes et al., 2010; Esper et al., 2016; Ljungqvist et al., 2020). The classical dendroclimatology presents vital information on tree growth–climate interactions, whereas more precise explanations of tree response to environmental variations are revealed by the studies of xylogenesis and resultant specific wood traits (De Micco et al., 2019).

Recent improvements in the methods of wood sectioning and technological advancement in measurement (semiautomatic and automatic) of cell anatomical traits have opened new possibilities in dendroclimatology (von Arx and Carrer, 2014; von Arx et al., 2016; Arzac et al., 2018; Peters et al., 2018; Gebregeorgis et al., 2021). For instance, quantitative anatomy of particular tree-ring zones from tracheidograms has emerged as one of the promising approaches (Vaganov et al., 2006; Carrer et al., 2017; Martin-Benito et al., 2017), which molds dendroclimatology of high temporal resolution on the scale of short intervals during the growing season (Pritzkow et al., 2014; Ziaco et al., 2016; Souto-Herrero et al., 2017; Pandey, 2021). However, statistical issues have been found in the utilization of QWA characteristics as new dendroclimatic indicators due to their possible correspondence both with climatic variables and among themselves, which is evident from numerous direct observations on seasonal tree-ring growth kinetics (Cuny and Rathgeber, 2016; Cuny et al., 2019; De Micco et al., 2019; Vieira et al., 2020; Stangler et al., 2021). In conifers, xylem development exhibits three main processes (i.e., production, cell growth by expansion, and deposition of the secondary cell wall) sequentially for each tracheid (Larson, 1994; Plomion et al., 2001; Fromm, 2013). The sequential order of cell production in the radial rows of tree-ring, as a result of cambial activity during the season and their maturation, provides information at a fine spatio-temporal scale for separation of external signal in resulting anatomical traits (Castagneri et al., 2017, 2018; cf. Carrer et al., 2017). However, possible regulatory connections between successive processes of xylogenesis and the degree of independence in their reactions to external factors are largely unknown and debated (Vaganov et al., 2011; Olano et al., 2012; Cuny et al., 2014; De Micco et al., 2019). Some studies consider each stage of xylogenesis as an independent recorder of the environment within the limits of its duration. Since the process of tracheid differentiation begins with the cambial zone (Larson, 1994; Plomion et al., 2001; Miyashima et al., 2013; Ramos and Regan, 2018; Aloni, 2021), other studies acknowledge cell division as the main receptor of external impacts, which conveys the received climatic signal to further stages of the differentiation of cells through intrinsic feedbacks (Vaganov et al., 2011, 2020). Such legacy effect links the magnitude and the timing of climatic factors at the time of tracheid division to their consequent development through the cambial zone. Its existence has been indirectly indicated by stable significant relationships found for morphometric parameters of the same tracheids or zones of tree ring among themselves and with cell production (Castagneri et al., 2017, 2018; Belokopytova et al., 2020; Piermattei et al., 2020; Zhirnova et al., 2021). Therefore, it is recommended to consider the presence of this legacy effect when attempting to use QWA in dendroclimatic analysis and reconstructions. Further, its removal from time series of tracheid anatomical traits can be performed using methods similar to those applied for the removal of long-term trends from tree-ring width measurements during their standardization (Cook and Kairiukstis, 1990). Particularly, we put forward modeling of the dependencies of anatomical traits on cell number in the ring (as characteristic of cambial activity) followed by indexation of anatomical chronologies in form of a ratio to the obtained model values. We suppose that such indexation may help separate climatic signals directly perceived by anatomical traits from the legacy of cambial activity reflected in cell number. The intrinsic relationships among wood traits can be best described using data set covering all or at least most of the life span of the tree, i.e., maximizing the length of anatomical chronology to decrease bias from age. However, due to a time- and resource-consuming nature, the studies in this field mainly operate with anatomical datasets lasting several decades or rarely reaching about a century (Yasue et al., 2000; Kirdyanov et al., 2003; Panyushkina et al., 2003; Castagneri et al., 2017). In this study, a long-term chronology (more than 200 years) of wood anatomical traits was developed with a primary focus to assess the relationships between measured wood characteristics. The main focus of the study was to reveal any existing relationships between cell number per ring and the characteristics of tracheidograms corresponding to the two subsequent phases of differentiation, and to recognize and quantify the shape of these relationships (linear or non-linear). This study was conducted with the following major objectives: (1) to determine whether cell radial size and wall thickness depend on the cell number per radial row of tree ring (indicating aforementioned legacy effect), (2) to express these dependencies analytically, (3) to develop indexed anatomical chronologies with eliminated legacy effect *via* division of actual values by modeled ones, and (4) to ascertain the separation of climatic responses in these “refined” anatomical chronologies.

## Materials and Methods

### Study Area

**Figure 1:**
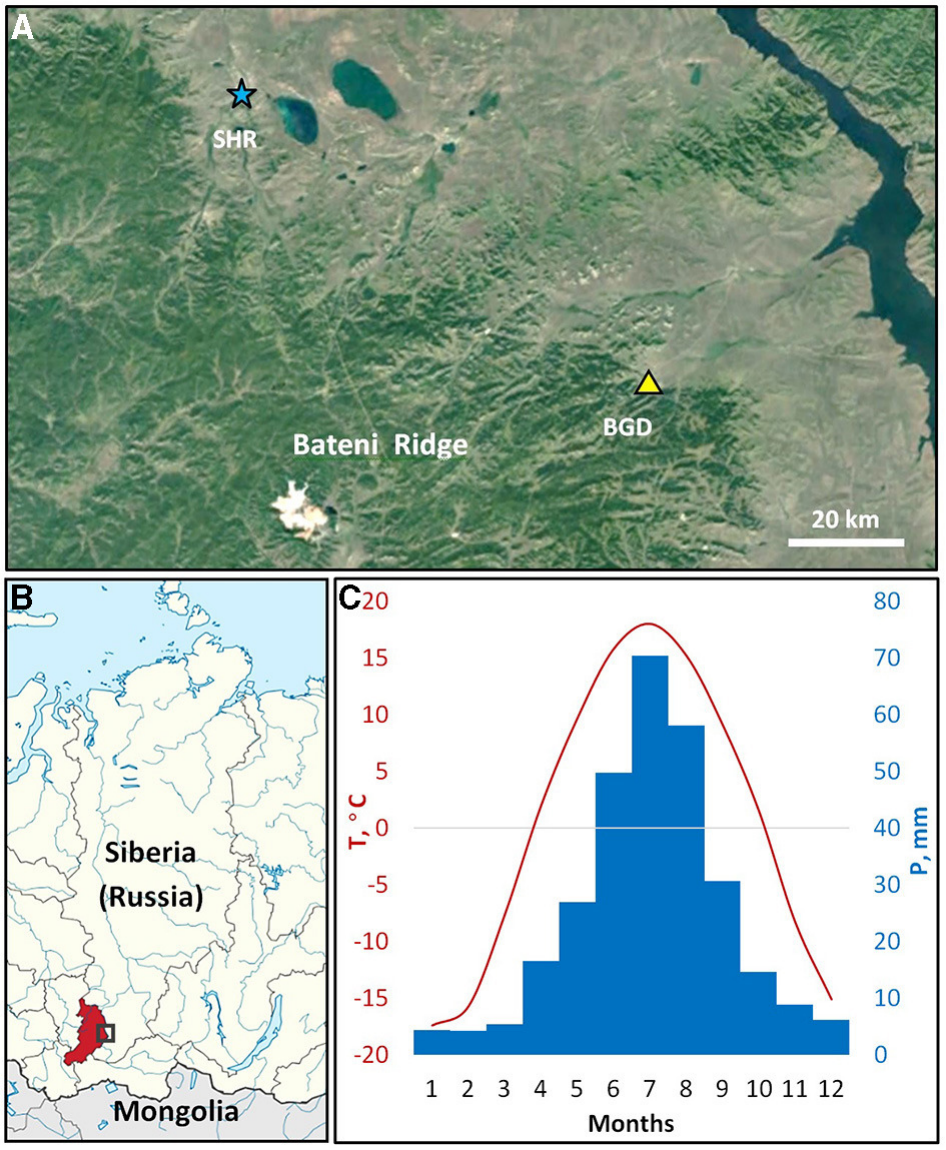
The study area. The territorial location of the sampling site (BGD; triangle) and the Shira meteorological station (SHR; asterisk) in the northern part of the Bateni Ridge, Kuznetsky Alatau mountain system **(A)**. The location of the study area (rectangle) in Siberia, Russia **(B)**; color marks administrative region, the Republic of Khakassia. Climatic diagram of average monthly temperatures (1966-2008; line) and precipitation (1936-2008, bars) at the Shira station **(C)**. Background maps: satellite data © 2020 Google; schematic map is fragment of Map of Khakassia within Russia (CC BY-SA 4.0).

The study was carried out in the eastern parts of the Bateni Ridge of Kuznetsky Alatau mountain range in southern Siberia (Figures 1A,B). The altitude of the ridge ranges from 500 m a.s.l. in the foothills to 1,200 m a.s.l. at the mountaintops in the central part. A mixed forest comprised of Siberian larch (*Larix sibirica* Ledeb.), Scots pine (*Pinus sylvestris* L.), and silver birch (*Betula pendula* Roth.) in different proportions occupies most of the ridge, but open forest stands alternating with steppe vegetation are found on the drier sites of southern and southeastern slopes, particularly in the foothills.

The region experiences a sharp continental climate (Alisov, 1956; Figure 1C) characterized by large seasonal and daily temperature drops. According to the closest meteorological station Shira (54.50° N 59.93° E, 475 m a.s.l.), the average annual temperature of the study area is ~+0.9°C with an annual precipitation of about 300 mm. As a rule, montane conditions witness lower temperatures and more abundant precipitation compared to plain areas, where Shira station is located. Precipitation of the area exhibited an uneven annual distribution with most of the rainfall (>80%) occurring during the season of positive temperatures, with the highest in July. The average frost-free period of positive daily minimum temperatures extends from May to September, where average daily temperatures are recorded above +5°C. Dendroclimatic analysis was performed using daily climatic series of precipitation (1936–2008) and temperature (1966–2008) from Shira station. Climatic data were obtained from the All-Russian Research Institute of Hydrometeorological Information, World Data Center (RIHMI-WDC; http://meteo.ru/data).

### Sample Collection, Measurement, and Processing of Tree Ring Data

Samples of Scots pine wood (cores) were collected from the vicinity of the Bograd village located at the northeastern tip of the ridge (BGD; 54.20–54.27° N 90.68–90.92° E, 500–650 m a.s.l.). The initial sampling was performed in 1989, which was subsequently repeated during 2012–2018. A total of 86 trees had been sampled during the whole period to find out the oldest pine trees in the area. Tree core samples were collected from healthy undamaged adult trees of the upper canopy level within the forest stand (tree height ~10–12 m), throughout slopes facing southern, southwestern, and southeastern aspects, where higher insolation increases the sensitivity of trees to moisture deficit and strengthens the climatic signal. Sample collection was performed using an increment borer at breast height (~1.3 m) perpendicularly to the slope, i.e., mainly in the west–east direction. The collected samples were transported to the laboratory and prepared for measurements as per the standard protocols laid down by Cook and Kairiukstis (1990). Tree ring width (*TRW*) series of each sample were measured using LINTAB-5 system and TSAP-win program (Rinn, 2003). The crossdating accuracy of the series was verified using COFECHA program (Holmes, 1983). Finally, a standardized local chronology of *TRW* was developed employing a computer program ARSTAN (Cook and Krusic, 2005). During the standardization procedure, long-term trends in individual *TRW* series were described by 67% cubic smoothing spline with a 50% frequency-response cutoff (Cook and Peters, 1981).

In 2019, seven cores were selected for anatomical measurements (one core per tree) out of the full sample collection. The cores were selected on the basis of maximum cambial age (the number of tree rings from bark to pith), high *TRW* correlations with the local chronology (Supplementary Figure 1), and their integrity. A rotary microtome (Microm HM 340 E; Thermo Fisher Scientific, USA) was used to obtain cross-sections of 12–14 μm thickness from selected tree cores excluding the areas of juvenile wood (the innermost 10–15 rings of notably different anatomical structure) to minimize height-related trends in wood hydraulic architecture (Mencuccini et al., 1997), if any was present. Cross-sections were stained with safranin and Astra Blue, dehydrated in increasing concentrations of ethanol, washed with xylene, and mounted in Canada balsam on glass slides.

**Figure 2:**
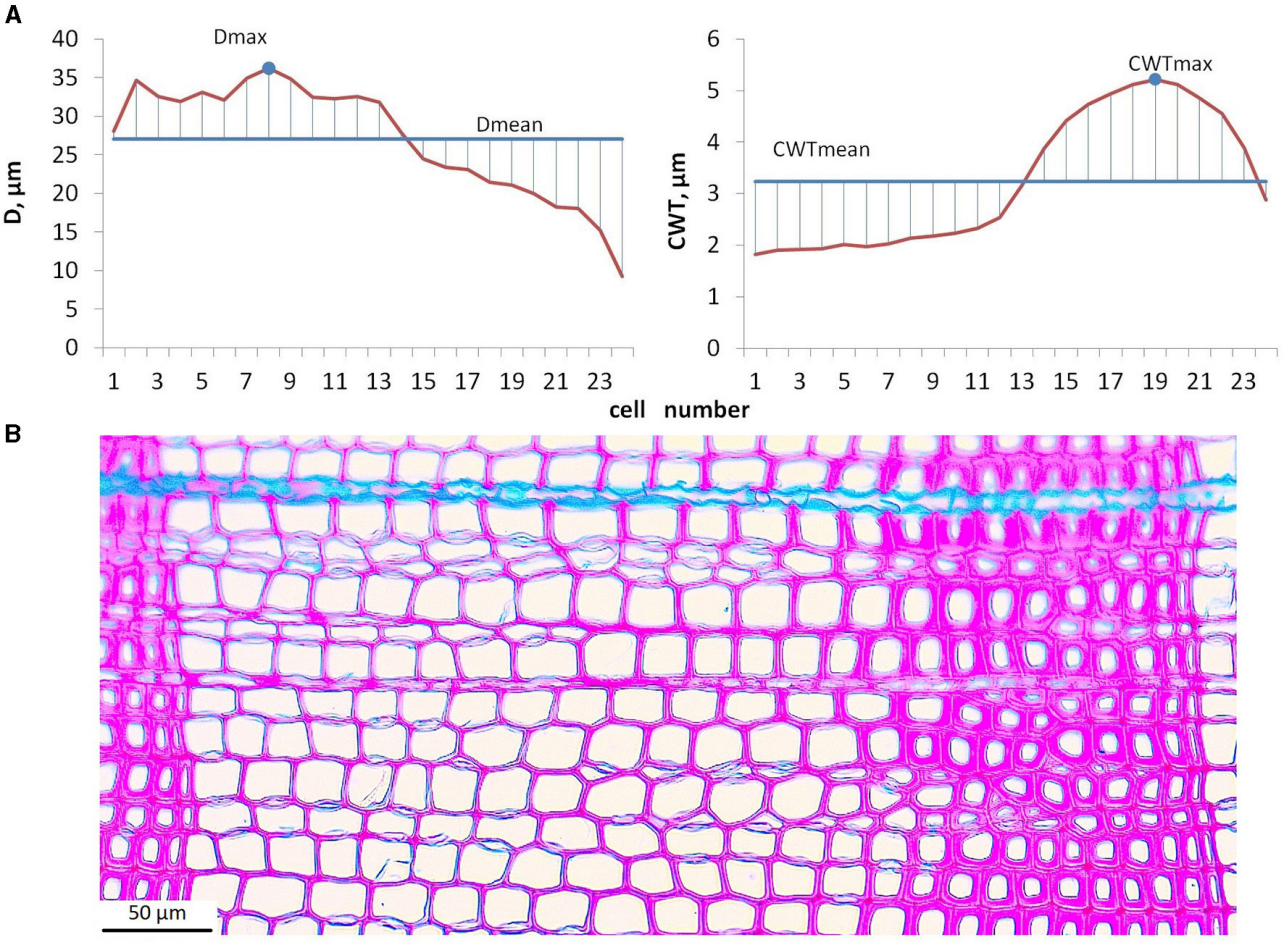
Calculation procedure for maximal and mean values of cell traits: averaged tracheidograms of radial diameter, *D* and cell wall thickness, *CWT* **(A)**; microphotograph of tree ring formed during 1903 in tree #6 used as an example for calculation **(B)**.

Microphotographs of cross-sections were obtained using a digital camera (ProgRes Gryphax Subra, Jenoptik GmbH, Germany) mounted on an optical microscope with 200x magnification (BX43, Olympus, Japan). Among the considered tree rings, numerous intraannual density fluctuations (IADF) have been observed in various positions within the ring. Some of the narrow rings showed thinner cell walls in latewood (light rings). Frost rings and blue rings were not observed. Anatomical traits of all the rings in the selected samples were measured using semiautomatic program Lineyka 2.01 (Silkin, 2010). Tree ring boundaries have been identified manually by the user. Wood anatomical traits, namely, the number of cells (*N*), their radial diameter (*D*), and cell wall thickness (*CWT*), were measured (Vaganov et al., 1985; Larson, 1994) for five radial rows of cells in each ring (Seo et al., 2014). To obtain the correct mean estimates of anatomical traits within each particular ring, intraseasonal *D* and *CWT* series (tracheidograms) were normalized by compressing/expanding them to the same number of cells as averaged over five measured radial rows (Vaganov, 1990). Due to the tediousness and the complexity of separating earlywood and latewood zones in numerous rings with IADFs, preference was given to the calculation of cell characteristics, not depending on the reliability of criteria used for zone separation. The cell characteristics, namely, maximum (*D*_max_) and average (*D*_mean_) radial diameter, and maximum (*CWT*_max_) and average (*CWT*_mean_) wall thickness, were calculated for each ring from normalized averaged tracheidograms (Figure 2). Robustness of these cell characteristics was tested by comparing mean values with median and maximum values with 90th percentile for a small subsample of randomly selected tree rings, both in individual radial rows of tracheids and in average tracheidograms (Supplementary Figure 2). Mean values of *D* and *CWT* integrate environmental signal during respective stages of cell development for all cells of tree ring in the same way as *N* assimilates environmental signal during cell division. Maximal values of *D* and *CWT* are closely related to mean *D* in earlywood and to mean *CWT* in latewood, respectively. Previous studies indicated that these relationships are stably linear for conifers in the study region (Zhirnova et al., 2021; unpublished data for Scots pine in various habitats), which is probably due to the suppression of stochastic outliers by averaging measurements of five radial rows in each ring. Correlations of maximal and mean values are high, 0.89–0.96 in individual trees and 0.95 in local average for *D*, and 0.81–0.94 and 0.74 for *CWT*, which means 66–92% of common variance. However, we expect even higher correlations between *D*_max_ and earlywood *D, CWT*_max_ and latewood *CWT* (for comparison, for Scots pine near the upper forest line in the study region the following correlations were observed: *r*(*D*_max_, *D*_mean_) = 0.85, *r*(*D*_max_, *D*_ew_) = 0.92, *r*(*CWT*_max_, *CWT*_mean_) = 0.89, *r*(*CWT*_max_, *CWT*_lw_) = 0.97; Zhirnova et al., 2021). Therefore, *D*_max_ and *CWT*_max_ can be interpreted as easily derived replacements of zonal characteristics, and they should register the environmental signal in more short-term intervals than *D*_mean_ and *CWT*_mean_, despite strong relationships between them.

Long-term chronologies of these four cellular characteristics were developed for every individual tree and finally averaged for all seven trees to obtain local chronologies. The omission of innermost rings minimized long-term tree size-related trends in anatomical series as no significant linear trends at *p* < 0.1 were observed either in the total series or in the earliest 50-year period. Therefore, detrending of anatomical chronologies was not performed. The individual *TRW* series were calculated as the sum of *D*. They were used to crossdate the anatomical measurement series with the preliminary LINTAB measurements of the same cores. The subseries of anatomically derived *TRW* for each core piece were separately crossdated with full *TRW* series measured on LINTAB (*r* > 0.9), and then, all the dated subseries were combined into one series. Additionally, these *TRW* series were crossdated with local *TRW* chronology (*r* > 0.7) developed from cores of all 86 trees collected at the sampling site (Supplementary Figure 1). Some rings in the chronology so obtained were absent as missing rings (a year without the tree growth due to unfavorable conditions). In several cases, the rings on boundaries of core pieces had become mechanically damaged when cutting and, therefore, were discarded at the measurement stage.

Legacy effect from the cambial zone in anatomical traits was assessed for the aforementioned generalized tracheid characteristics. Cell number *N* was interpreted here as resulting characteristics of cambial activity aggregated for all vegetative season. Similarly, mean tracheid measurements were used as aggregated resulting characteristics of the consequent xylogenesis stages. Albeit production and differentiation of the largest cell and of the most thick-walled cells in tree ring have to be confined to the certain smaller time intervals within the vegetative season, there is no detailed information available on cell production during these intervals; thus, these maximal values were also compared with the total cell number. Dependencies were analytically described by non-linear regression models *D*_max_(*N*), *D*_mean_(*N*), *CWT*_max_(*N*), and *CWT*_mean_(*N*) using equation and numerical terms aiming for better statistical fitness of models (maximizing *R*^2^) and for easy ecological interpretation. The models were derived using a unified form of the equation, whereas numerical terms were developed separately for each considered tree due to expected differences in requirements to water conductivity and mechanical strength of xylem owing to the allometry of trees, genetic predisposition, and microconditions (Mencuccini et al., 1997; Anfodillo et al., 2012, 2013). Elimination of legacy effect was performed by calculating indexed series (the ratio of actual to modeled values) for each tree and averaging them to obtain local chronology. This approach provided better results of further analyses (closer to normal distribution of indexed values, higher common signal, and climatic correlations for local chronologies) in comparison to subtraction of modeled values from actual ones (data not presented).

Climatic response of averaged local chronologies of *TRW* and indexed anatomical parameters was estimated in the form of correlations with moving series (21-day window, 1-day step within season) of temperature and precipitation, which were calculated using daily data from the Shira station. Analysis was performed for the entire cover period of climatic data and for moving 30-year intervals (1-year step) to assess the temporal stability of response.

## Results

**Figure 3:**
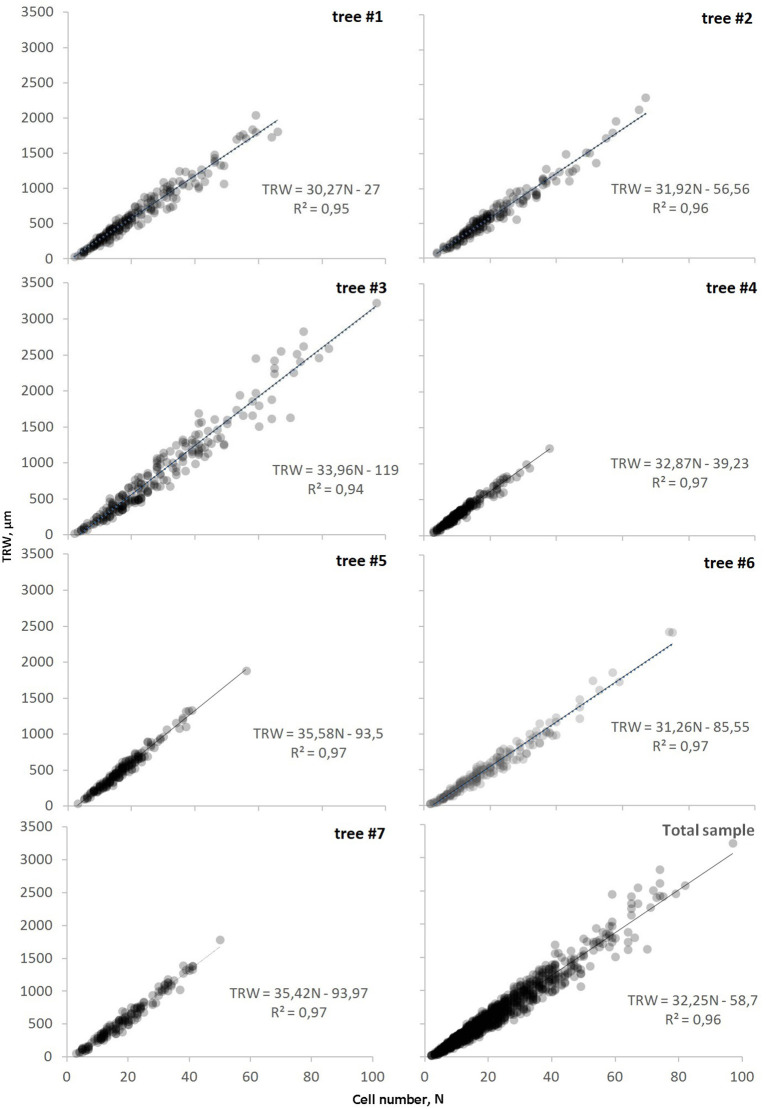
Dependencies of tree-ring width on cell number, *TRW*(*N*), for each investigated tree and on the scale of sampling site (total sample). Markers (circles) represent actual tree rings and line represents the linear function of relationship (equation and coefficient of determination R^2^ is shown on each plot).

The length of the individual series of anatomical measurements ranged from 150 to 280 years spanning over the period 1739–2018 CE (Supplementary Figure 1). The classical tree-ring width parameter showed a linear dependence on the cell numbers through linear regression models *TRW*(*N*) (Figure 3). The relationship was almost directly proportional, as marked by the value of the constant term not exceeding 120 μm and being non-significant at *p* < 0.05. The widest rings containing the largest number of cells were observed in tree #3 with a cambial age of 222 years, whereas the oldest sample (tree #4, 280-year-old) exhibited the least variability of tree-ring width and cell numbers (Supplementary Table 1). Nevertheless, no significant relationships were found between the cambial age of trees and the range of cell numbers in the ring. For all the samples, the coefficient of determination was close to the maximum (0.95–0.98) and indicated the high reliability of the obtained models along with their strict proximity to a linear relationship. The visual comparison of graphs summarizing information on all the modeled trees illustrated the near-linearity of the *TRW*(N) on the local scale too. The majority of rings (95%) were found in the range of *TRW* < 1,400 μm and *N* < 45.

**Figure 4:**
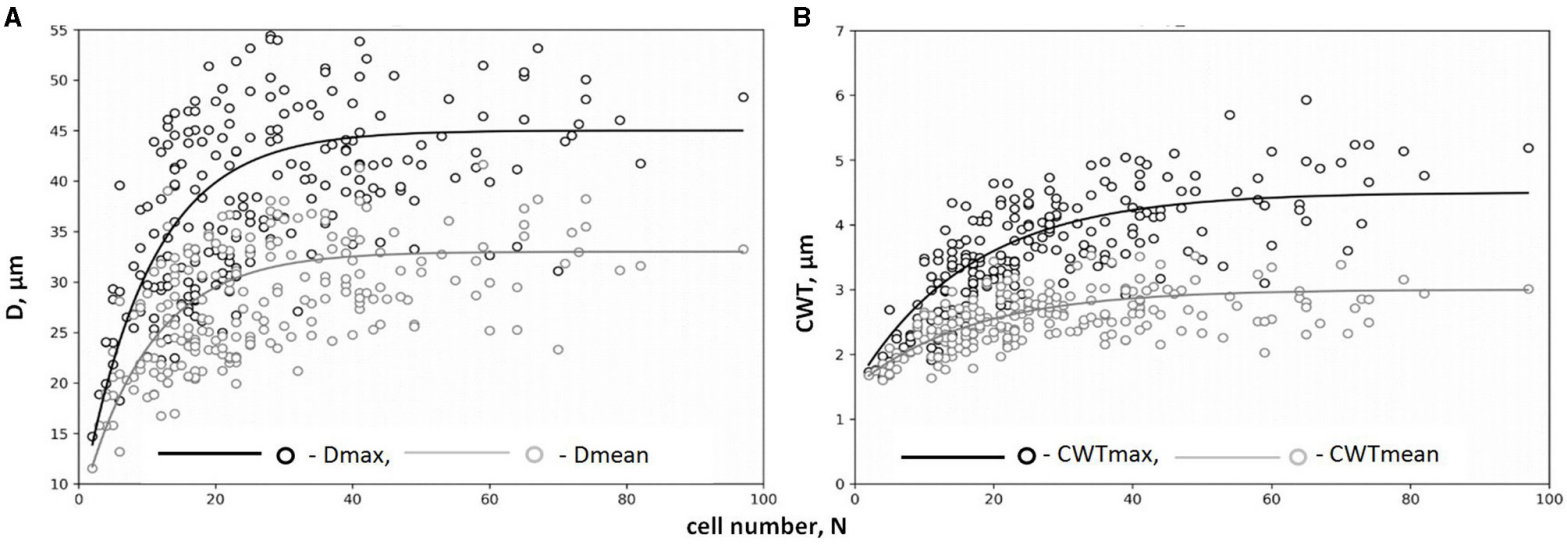
Dependencies of tracheid traits on cell number, *N*, for maximum and mean values of radial diameter *D*_max_(*N*) and *D*_mean_(*N*) **(A)** and cell wall thickness *CWT*_max_(*N*) and *CWT*_mean_(*N*) **(B)** (on example of tree #3). Markers of scatter plots (circles) represent actual tree rings; lines represent exponential functions of relationships. Data on maximum values of cell traits (*D*_max_, *CWT*_max_) are presented in black color; data on mean values of cell traits (*D*_mean_, *CWT*_mean_) are presented in gray color.

The legacy effect of cell production in QWA was characterized by the dependencies of tracheid characteristics on the cell numbers and well described by a negative exponential function (Figure 4, Supplementary Figure 3). A rapid increase in both mean and maximal values of *D* and *CWT* per tree ring was observed in narrow rings synchronously to an increase in *N* but gradually turned to stable values of cell characteristics in wider rings with large *N*. After selecting the numerical parameters for all exponential models (Table 1), the correlations between model values and actual measurements ranged from 0.53 to 0.86.

**Table 1:** Numerical parameters of the dependencies of tracheid measurements (cell radial diameter D and cell wall thickness *CWT*) on cell number *N*, expressed as functions $D\;=\;D_{\min}+\left(D_{as}-D_{\min}\right)\cdot \left(1-e^{-a\cdot N}\right)$, $\text{CWT}\;=\;{\text{CWT}}_{\min}+\left({\text{CWT}}_{as}-{\text{CWT}}_{\min}\right)\cdot \left(1-e^{-a\cdot N}\right)$ fitted for maximal (*D*_max_, *CWT*_max_) and mean (*D*_mean_, *CWT*_mean_) values in the ring.

**Tree**	***D***_**max**_				***D***_**mean**_			
	***D*_**min**_, μm**	***D*_**as**_,** **μm**	***a***	*R* ^*^	***D*_**min**_, μm**	***D*_**as**_,** **μm**	***a***	***R***
tree #1	7	43	0.17	0.59	7	32	0.12	0.54
tree #2	7	43	0.14	0.48	7	32	0.12	0.53
tree #3	7	45	0.10	0.61	7	33	0.10	0.59
tree #4	7	41	0.25	0.69	7	31	0.22	0.66
tree #5	7	45	0.13	0.78	7	34	0.13	0.73
tree #6	7	43	0.11	0.74	7	30	0.11	0.74
tree #7	7	46	0.14	0.71	7	32	0.15	0.71
**Tree**	***CWT***_**max**_				***CWT***_**mean**_			
	***CWT***_**min**_, **μm**	***CWT***_**as**_, **μm**	***a***	***R***	***CWT***_**min**_, **μm**	***CWT***_**as**_, **μm**	***a***	***R***
tree #1	1.5	4.0	0.07	0.72	1.5	2.6	0.12	0.54
tree #2	1.5	4.4	0.045	0.81	1.5	3.0	0.04	0.76
tree #3	1.5	4.5	0.06	0.79	1.5	3.0	0.06	0.63
tree #4	1.5	4.5	0.06	0.69	1.5	3.5	0.06	0.41
tree #5	1.5	4.7	0.055	0.69	1.5	3.0	0.05	0.58
tree #6	1.5	5.7	0.05	0.85	1.5	3.3	0.06	0.74
tree #7	1.5	4.3	0.05	0.86	1.5	2.8	0.04	0.80

* *R, correlations between modeled and actual series*.

*Equations are written so cell measurements tend to minimal values D_min_, CWT_min_ for extremely narrow rings (N → 0) and to asymptotic values D_as_, CWT_as_ in the area of wide rings, a is numerical term of exponential function regulating curve flexibility*.

**Figure 5:**
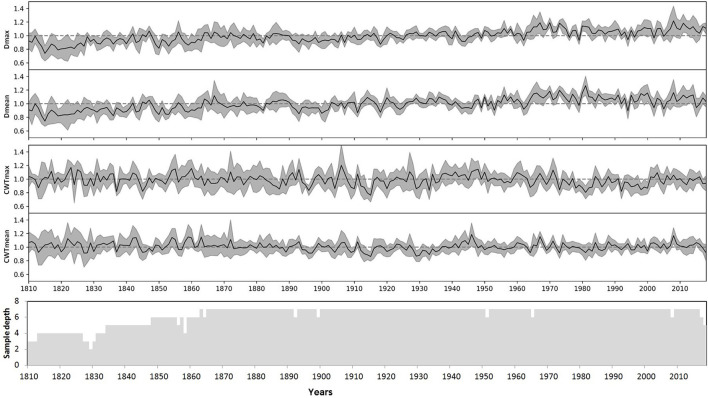
Local indexed chronologies of anatomical characteristics of the Scots pine: maximum (*D*_max_) and mean (*D*_mean_) values of cell radial diameter, maximum (*CWT*_max_) and mean (*CWT*_mean_) values of cell wall thickness in the tree ring. Lines represent local average chronologies; shaded areas represent standard deviations for each calendar year. The bottom panel shows the dynamics of the sample depth (number of individual series), taking into account missing and discarded rings.

The indices for each tree were calculated as a ratio of the actual measurements to the respective model values, an approach similar to the *TRW* indexing in classical dendrochronology. The indexed values have small skewness and kurtosis, and similar values of the median and arithmetic mean for most characteristics and individual trees (Supplementary Figure 4, Supplementary Table 2). Finally, local chronologies of considered anatomical traits were calculated by averaging the individual indexed series. Local chronologies covered a period of 279 years, whereas the sample depth of at least three trees was obtained for 211 years (1807–2018), and five trees covered a period of 185 years (1834–2018) (Figure 5). Chronologies of *CWT* characteristics have been stable with time, whereas indexed chronologies of both *D*_max_ and *D*_mean_ exhibited slowly increasing trends. For the period 1807–2018, the slopes of these trends were about 0.001 relative unit per year with coefficients of determination *R*^2^ = 0.57 and *R*^2^ = 0.48 at *p* < 0.01 for chronologies of *D*_max_ and *D*_mean_, respectively. Nevertheless, for periods of dendroclimatic analysis, *R*^2^ < 0.2 was observed for linear long-term trends in all four chronologies, and long-term variation was much smaller than year-to-year fluctuations.

It was noted that the initial average inter-serial correlations (0.36–0.40) between the anatomical chronologies of seven individual trees substantially decreased after indexing (*D*, 0.26–0.30; *CWT*, 0.11–0.13). Furthermore, the average correlations of individual series with respective local chronologies were recorded as 0.67–0.70 before indexing for all cell characteristics, 0.64–0.66 for indexed *D*, and 0.51–0.55 for indexed *CWT*.

**Figure 6:**
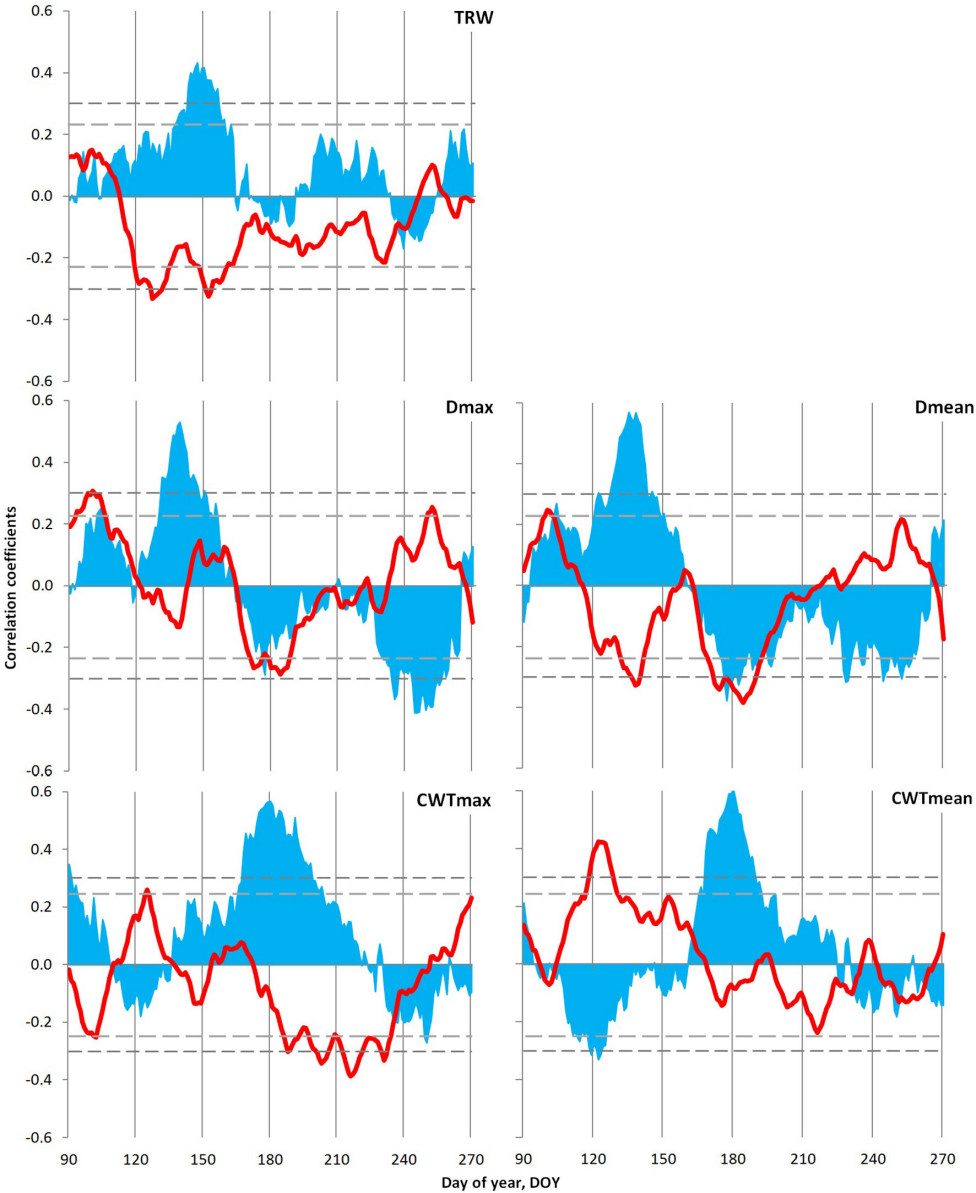
Climatic response of tree ring characteristics of the Scots pine: tree ring width (*TRW*), maximum (*D*_max_) and mean (*D*_mean_) values of cell radial diameter, and maximum (*CWT*_max_) and mean (*CWT*_mean_) values of cell wall thickness in the tree ring. Plots show correlations of indexed local chronologies with moving series of temperature (red lines; 1966–2008) and precipitation (blue areas; 1936–2008), calculated from daily data with a 21-day window and 1-day step. The horizontal dashed lines show the significance level *p* = 0.05 for correlations with temperature (short dashes) and precipitation (long dashes).

Tree growth–climate relationship for local indexed chronologies was analyzed using daily data of temperature (1966–2008) and precipitation (1936–2008) generalized with a window of 21 days. Significant relationships have been observed between local indexed chronologies and climate variables during the season from 90 to 270 day of the year, i.e., March 30–September 26 (Figure 6). The *TRW* of Scots pine correlated positively with precipitation and negatively with temperatures from early May to mid-June. On the contrary, a different pattern has been observed with cell diameter (*D*_max_ and *D*_mean_), where humid conditions during May to June favored the formation of large cells, and temperatures of this period displayed a significant negative relationship with *D*_mean_. From mid-June to the end of July, a negative influence of both climatic variables has been detected on cell size, which was more pronounced in the case of *D*_mean_. Moreover, *CWT* revealed a dependence on precipitation from mid-June to early August, whereas a negative association of air temperature was more pronounced with *CWT*_max_ and observed during July–August. However, a positive link between *CWT*_mean_ and temperatures in May has added an interesting fact in the case of the tree growth–climate relationship of Scots pine.

Temporal stability analysis of the climatic response of Scots pine *TRW* chronology (Supplementary Figure 5) exhibited a decrease in response intensity. On the contrary, both maximal and mean anatomical traits of tree rings registered gradual shifts in the intervals of significant climatic signal within the season, but its intensity remained stable. Both *D*_max_ and *D*_mean_ showed maximum positive reaction to precipitation during the earlier dates of the summer season recently, whereas the period of their negative reaction to temperature and precipitation remained fixed during the second half of summer. On the contrary, *CWT* displayed a positive response to moisture for a prolonged period (the beginning date for significant correlations stably coincides with the summer solstice, combined with the later ending of the reaction in the last decades) with a shift in negative response to temperature to the later dates.

## Discussion

### Legacy of Cell Production in Anatomical Traits of Tree Ring

The tree-ring width as the sum of the cell radial diameters assimilates the results of two successive stages in tracheid differentiation, namely, cell division in the cambial zone and their subsequent growth by expansion during the season of growth (Larson, 1994; Fromm, 2013; Rathgeber et al., 2016). Therefore, any deviations of the *TRW*(N) function from direct proportionality (i.e., a linear function with a zero constant term) were caused by the variability of the average *D* in the tree ring. This variability is made up of several components: (i) age-size trends in juvenile wood (Vysotskaya and Vaganov, 1989; Lei et al., 1996; Anfodillo et al., 2013) omitted in the considered dataset, (ii) observed legacy effect described by an exponential function *D*(*N*), (iii) impact of current climatic fluctuations during cell expansion, and (iv) stochastic component. Consequently, *TRW*(N) function exhibits a slight curvature in the area of narrow rings (Figure 3). Nevertheless, the very high coefficients of determination obtained for linear models indicated high stability of the average size of cells produced near the base of the tree trunk during most of the lifespan of the tree. On the contrary, the proximity of this relationship approved *TRW* as a parameter integrating the influence of climatic fluctuations mainly during the period of cambial activity and before its beginning (through the state of the tree at the moment of cambium activation). Although weather fluctuations affect the anatomical structure and functional traits of the xylem produced during the ensuing stages of tracheid differentiation after the end of cell division, their effect was not practically reflected in the *TRW*.

Anatomical traits revealed lesser variability with a limited range of values as compared to cell production, which is mainly linked with the functional limitations of the xylem as a mechanical and water-conducting tissue (Fromm, 2013). However, the functional ability of the xylem is sensitive to even a small variation in cell size (e.g., the ability to conduct water depends on the fourth degree of the lumen diameter; Tyree and Ewers, 1991). Such functional limitation can certainly complicate the relationships of anatomical characteristics with other tree-ring traits and with climatic variables. Dependence of anatomical characteristics of tracheids on their production has been observed for all trees and displayed a common and obviously non-linear pattern. This non-linearity can be expressed through a significant positive relationship observed between production and subsequent differentiation processes in case of a small number of cells, where stress conditions during cell division are translated into the formation of only a few small and thin-walled tracheids. However, the greater cell production weakens this relationship, and the widest rings formed under favorable conditions have anatomical traits practically independent of cell production.

In wide rings, both the average and maximum estimates of considered cell parameters fluctuate around values that probably reflect optimal functionality of the xylem for every individual tree. The differences in these values between trees express the genetic or phenotypic (tree height, dimensional characteristics of crown, and root system) setting of a particular tree and micro conditions of habitat. The assumption about partial genetic conditionality of the xylem structure has been confirmed by scales of species and provenances by several studies, e.g., quantitative analysis of anatomical traits for pines of different provenances sharing habitat (Martín et al., 2010; Matisons et al., 2019). Dependence of QWA traits on species was observed on an individual tree scale comparing scion and rootstock tree-ring structure in the same year within heterografts of Siberian pine on Scots pine (Darikova et al., 2013). Obviously, the genetic-driven difference occurs on the smallest scale of individual genotypes too. The dimensional characteristics of the tree, particularly its height, the proportion of fine roots, and the photosynthetic apparatus determine the demand of the plant for water supply and yield the previously described trends in anatomical structure along its vertical and radial gradients, as reported by Anfodillo et al. (2012, 2013) and other researchers (Vysotskaya and Vaganov, 1989; Lei et al., 1996; Mencuccini et al., 1997). However, in this study, the influence of tree-height dynamics has been minimized, which is indicated by the absence of long-term trends in the measured cell characteristics, and therefore, only the difference in the final height of mature trees matters. Microconditions determine the variation of microclimate, availability of resources for tree growth and development, and resource competition with neighbors (Jaques, 1995; Van Do et al., 2015), which also quantitatively affect the optimal structure of wood. However, it is noteworthy that differences between individuals have not affected the fundamental character of D(N) and *CWT*(N) dependencies. All cells of the nascent xylem at the time of their production presented the size and the primary *CWT* similar to those of the cambium, which provided the opportunity to describe the revealed dependencies in the form of exponential functions with numerical coefficients of explicit values. Extreme external conditions suppress the rates of all growth processes (cell division and differentiation) in the xylem simultaneously. Assuming a common zero point for these rates of growth processes, the zero cell division will correspond to zero expansion and zero secondary wall deposition. Therefore, at the points of the intersection of functions with the vertical axis (the theoretical limit N → 0), tracheids should have the same measurements as initial cambial cells not passed through the processes of differentiation. The asymptotic values *D*_as_ and *CWT*_as_ to which the curve tends in the region of wider rings (*N* → max) characterized the balance between efficiency and safety of optimal hydraulic architecture (lumen size and tracheid strength) for each tree. The numerical coefficient (*a*) regulates the range of substantial dependence of anatomical characteristics on cell production.

### Obtained Long-Term Indexed Chronologies as Independent Proxies of Climate Impact on Tree Growth

The removal of legacy effect by dividing the actual measurements with model values, a procedure similar to standardizing the tree-ring width chronologies, resulted in obtaining the indexed chronologies for anatomical traits. Values of these indexed chronologies were closer to a normal distribution as compared with the initial measurements and, therefore, displayed more suitability for dendroclimatic analysis. Although presented models of dependencies on *N* exhibit signs of imperfection, at least for cell diameter characteristics, since their indexed chronologies have subtle long-term trends absent in measured values. Further studies on the long-term stability of variation in anatomical chronologies are required to improve this simple approach of indexation so that they can be used as multicentennial proxies for dendroclimatic reconstructions.

Indexed chronologies have been developed with a lower amount of common signal due to the elimination of variation inherited from cell production and extrinsic (climate) processes recorded in it. However, significant relationships of these chronologies were still observed with climatic factors. The independence of the indexed anatomical chronologies from cambial activity processes has been reflected by drastic differences in their response to climate as compared with climatic signals registered in *TRW*, which is closely related with *N*. Henceforth, a set of standardized chronologies have been obtained that record the impact of climatic fluctuations during the season separately and independently on all three stages of xylem formation, i.e., (1) *TRW* as an indicator of the impact of environmental conditions on cell production in the cambial zone; (2) *D*_max_ and *D*_mean_, explaining climatic conditions during the process of cell expansion in the middle of earlywood zone and the entire ring, respectively; (3) *CWT*_max_ and *CWT*_mean_, indicating climate variability during the process of deposition of the secondary cell wall in the typical latewood tracheids and the entire ring, respectively.

Comparison of responses of developed chronologies to climate variables revealed distinct differences in seasonality, intensity, and even the direction of the effects of temperature and precipitation on the successive stages of wood development. Cell production showed a climatic response to moisture availability typical to semiarid regions, where positive correlations indicate precipitation as a source of water, and negative correlations mark the temperature as a drying factor (Liu et al., 2013; Bhandari et al., 2020). However, at least for the pine of the study area, it has to be remembered that large-sized earlywood cells make a major proportion in terms of cell numbers. This might be linked with the species-specific predisposition and with the climate type of the study area. In the continental climate, the cambial activity rate exhibits unimodal and asymmetrical kinetics with a maximum closer to the onset of the season coinciding with earlywood development (Popkova et al., 2018; Fonti et al., 2020), which lowers the latewood proportion for Scots pine to about one-third of the ring as observed in Scots pine trees growing in various natural zones of the region (Zhirnova et al., 2020, 2021). Therefore, the *TRW* climatic response was more focused on earlywood than latewood, and seasonal interval of significant correlations (i.e., May to the first half of June) has been consistent with the production of earlywood tracheids. The differences in the seasonality of climatic response of earlywood and latewood have been repeatedly noted by different researchers in their studies of earlywood and latewood widths (De Grandpré et al., 2011; Crawford et al., 2015; Acosta-Hernández et al., 2019). These patterns are also applicable to cell production in these zones, for obvious reasons.

The maximum cell size in the ring depends on the amount of precipitation soon after the beginning of the growing season when water availability regulates the turgor and determines the rate of expansion that results in the formation of the largest tracheids occurring during this time (Passioura and Fry, 1992; Cabon et al., 2020; for the typical position of the largest cell in the middle of earlywood see Figure 2). Also, large tracheids of this zone combine intensive rates and long duration of cell expansion (Anfodillo et al., 2012; Cuny, 2013). Thus, even if *D*_max_ is originated from the measurement of only one cell, its value in the particular ring might register water availability during several weeks. Year-from-year shifts in dates when the largest cell is being expanded provide a further increase of intraseasonal interval covered by the climatic signal in *D*_max_ long-term chronology to about the length of a month. For the average cell size, a similar response to early summer conditions paved the way to negative correlations with both climatic factors at a later time in the season, which can be explained by several reasons. For example, a negative response to precipitation can be associated with a larger proportion of small latewood cells in the ring produced during wetter conditions. This hypothesis has been supported by clear temporal stability observed for the onset of this particular response that was fixed near summer solstice as a trigger factor for latewood formation (cf. Drew and Downes, 2015, and references there). On the contrary, an increased temperature can be considered as a stimulant to plant development by reducing the duration of certain stages. Even the timing of various phenophases for many cultivated plants has been calculated using the sums of active temperatures (Grigorieva et al., 2010; Hatfield et al., 2011; Wypych et al., 2017). Since the length of the cell expansion process contributes more to the final radial size than its rate (Anfodillo et al., 2012; Cuny, 2013), an increase in temperature coupled with sufficient humidity can lead to a decrease in cell size due to a faster transition of cells to the stage of secondary wall deposition.

A positive response of the *CWT* to temperatures and a negative response to precipitation during May were observed in *CWT*_mean_ but not in *CWT*_max_, which can be interpreted as earlywood being a recorder of this signal. The formation of smaller and thicker earlywood cells under dry and hot conditions could be the part of the plant adaptation mechanism of the earlywood structure toward drought by providing a higher safety of water supply (reducing the risk of cavitation and embolism) *via* redistribution of available resources from cell expansion to wall deposition. This phenomenon during the most severe droughts leads to the formation of IADF in the form of a band of latewood-like tracheids in earlywood (Lautner, 2013; Battipaglia et al., 2016; De Micco et al., 2016). However, the requirement for sufficient mechanical strength of the tracheid walls of smaller cells of latewood usually met without the presence of such a reaction.

In contrast to pine stands growing in the center of the valley, completely within the arid steppe zone with possible genetic adaptation to frequent droughts, the sampled population of mountain forest in this study could be adapted to any particular climatic extreme only through phenotypic plasticity, due to the mixing of the genetic pool between trees growing at a variety of elevations and slopes (cf. Alden and Loopstra, 1987; Larionova et al., 2007; Chung et al., 2020). Therefore, like other mountainous habitats of the region (see Belokopytova et al., 2020), the *CWT* in the latewood expressed a positive reaction to precipitation and negative feedback with temperatures toward the end of the season, as reflected in both *CWT*_max_ and *CWT*_mean_ chronologies.

Regional warming in the study region has obviously increased the length of the season with temperatures appropriate for wood formation, both in spring and in autumn (see Rogers and Mosely-Thompson, 1995; Roshydromet, 2014). It is very expository that indexed anatomical chronologies responded much more clearly and distinctively to this shift than *TRW* chronology, as revealed from the temporal stability analysis of climatic responses. *D* chronologies indicated a clear earlier beginning of xylogenesis, whereas the ending point of significant dendroclimatic correlations for *CWT* shifted to the later calendar dates. At the same time, patterns of response in the middle of the season representing a transition from earlywood to latewood staid “glued” to the same calendar date in the second half of June, which adds evidence for daylength being the main driver of this switch for Scots pine. Overall, stable intensity and uniform long-term shifts of climatic signal in the indexed anatomical chronologies support their usage as fine-scale climatic proxies, despite lowering of the common signal during indexation.

## Conclusions

In this study, while tree-ring width exhibited linearity of almost direct proportion to cell numbers, cellular traits deciphered non-linearity of hydraulic and mechanical functions of xylem with regard to cell production and displayed a stronger relationship in the areas of narrow rings and stable anatomical structure of wider rings. These relationships were described by a negative exponential function. Furthermore, climate–growth relations revealed that standardized indexed chronologies of wood traits are strongly influenced by climatic factors, especially moisture availability. This reflects the applicability of this study on plant growth and wood quality in changing climate scenarios, particularly droughts. However, specific mechanisms and the causal nature of the observed climatic reactions need to be further explored in terms of tree physiology of this and other species to obtain a more robust database to make a strong observation. The temporal stability of revealed relationships also has to be explored on a centennial scale, as the length of the presented dataset allows such investigation. Regardless of the direction of future research, the obtained climate-sensitive anatomical chronologies in this study offer an important trait of distinct temporal intraseasonal separation of the climatic signal. Increasing the cover period for chronologies of used anatomical datasets constitutes them as promising proxies for the inverse dendroclimatic task, which can lead to the reconstruction of important climatic factors for relatively short intraseasonal intervals.

## Data Availability Statement

The original contributions presented in the study are included in the article/Supplementary Material, further inquiries can be directed to the corresponding author.

## Author Contributions

EV conceived and designed the research. EB supervised and coordinated study in general. EV and EB provided financial support. EV, EB, DZ, and LB performed fieldwork. DZ supervised and participated in the processing of samples, and supervised anatomical measurements. DD and MZ under guidance of LB performed calculations, data analysis, and created graphics. LB, KU, and ST finalized the manuscript and later revised it. All authors contributed to interpretation of results and writing of the first draft.

## Funding

This research was funded by the Ministry of Science and Higher Education of the Russian Federation, scientific topic code FSRZ-2020-0010, and the Russian Foundation for Basic Research, grant number 20-016-00049. The funding sources had no involvement in study design, in the collection, analysis, interpretation of data, in the writing, and in the decision to submit the article for publication.

## Conflict of Interest

The authors declare that the research was conducted in the absence of any commercial or financial relationships that could be construed as a potential conflict of interest.

## Publisher's Note

All claims expressed in this article are solely those of the authors and do not necessarily represent those of their affiliated organizations, or those of the publisher, the editors and the reviewers. Any product that may be evaluated in this article, or claim that may be made by its manufacturer, is not guaranteed or endorsed by the publisher.
