# Supplementary material for: Non-linear Response to Cell Number Revealed and Eliminated From Long-Term Tracheid Measurements of Scots Pine in Southern Siberia
Source: Front Plant Sci. 2021 Oct 4;12:719796. doi: 10.3389/fpls.2021.719796 (PMC8521138; doi:10.3389/fpls.2021.719796)
Supplement: Supplementary file 1 [file Data_Sheet_1.PDF]

# *Supplementary Material*

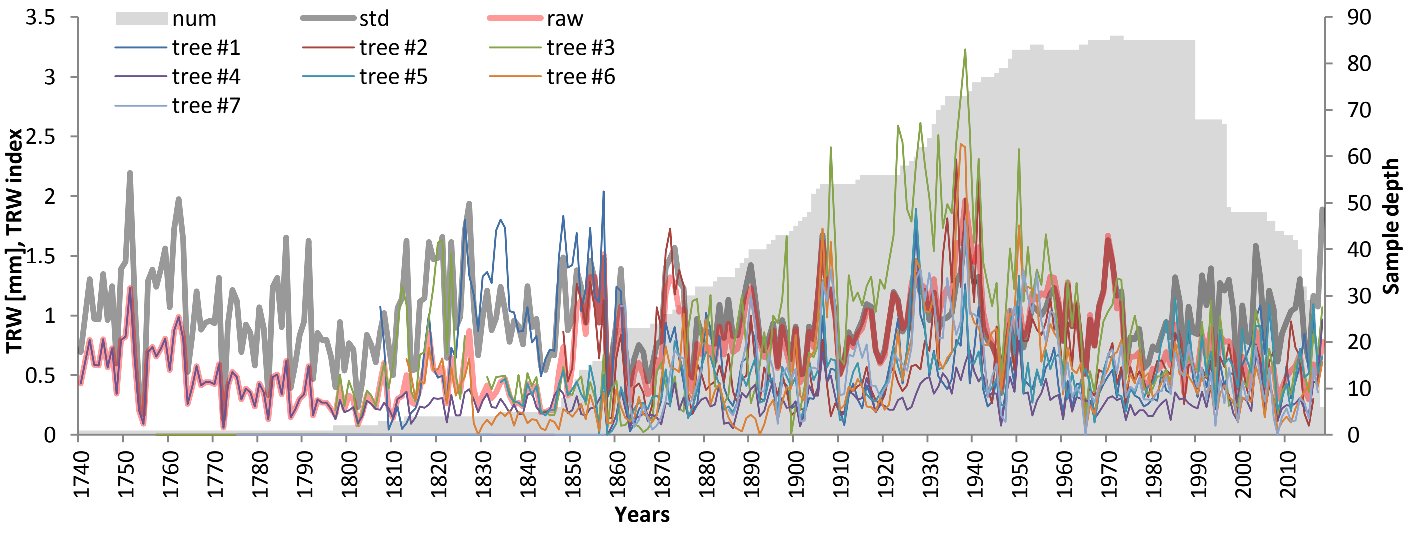


**Supplementary Figure S1.** TRW series of individual trees calculated from anatomical measurements (thin lines). For comparison, local chronology averaged from 86 cores is presented (red thick line, raw – measured chronology; dark grey thick line, std – standard chronology; light grey area, num – dynamics of core number)

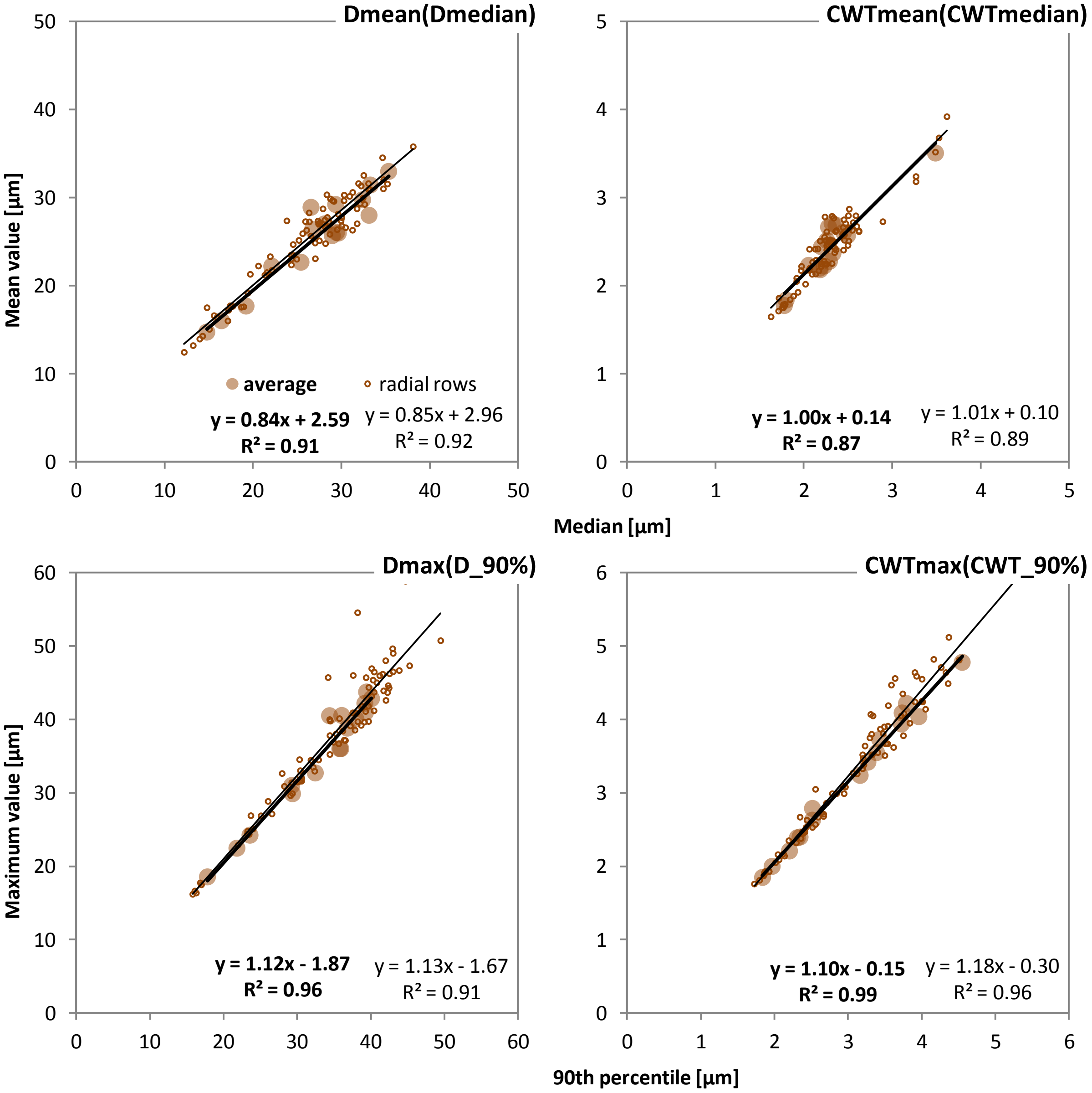


**Supplementary Figure S2.** Scatter plots comparing mean and median values (top row), maximum and 90th percentile values (bottom row) of radial cell diameter D (left column) and cell wall thickness CWT (tight column) for subsample of 14 tree rings (2 randomly selected rings per tree). Small dots represent data of five measured radial rows of tracheids; large dots represent average tracheidograms of each tree ring. Thin lines / normal text and thick lines /bold text represent respective linear regressions

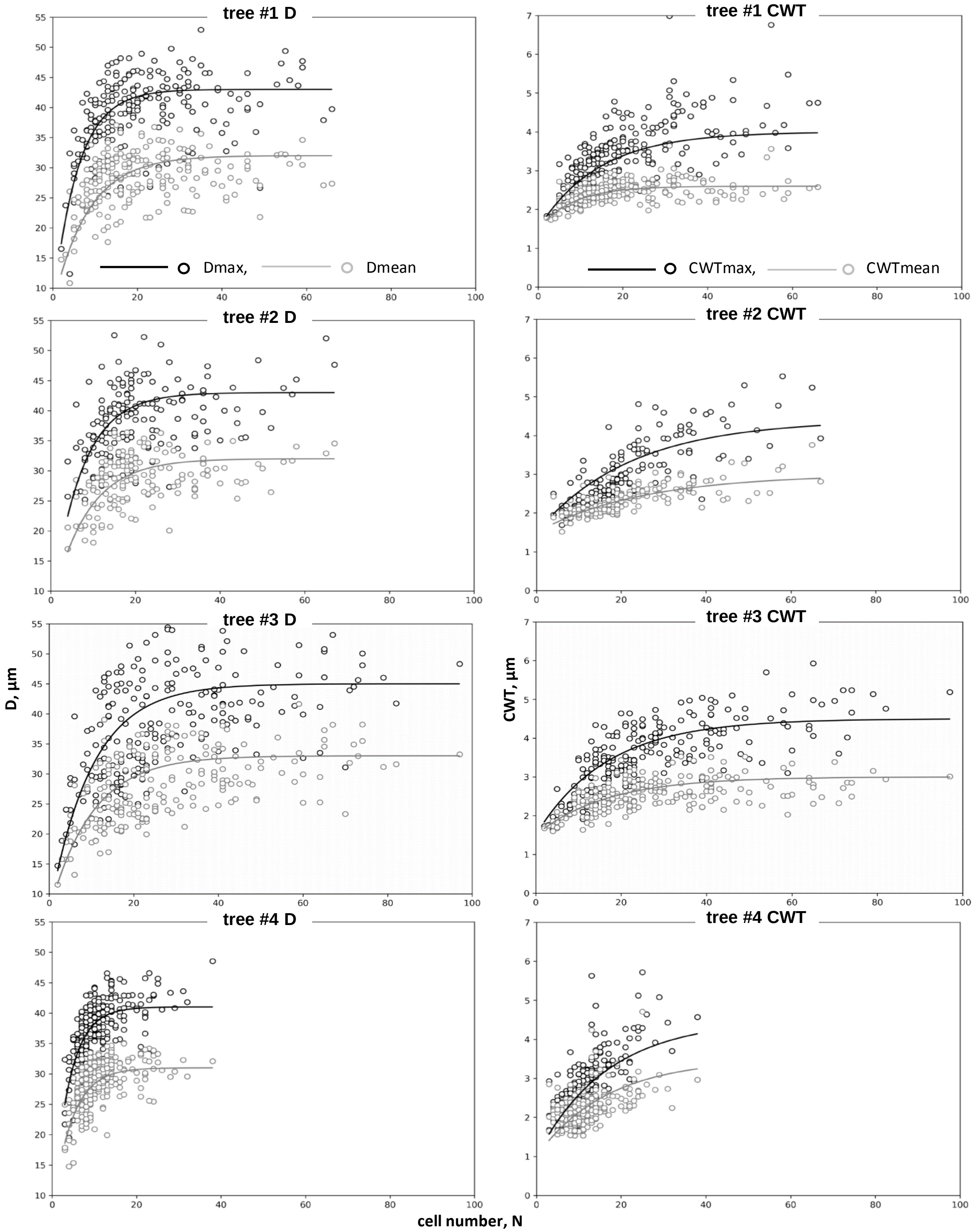


**Supplementary Figure S3.** Dependences of tracheid traits on cell number N for maximum & mean values of radial diameter Dmax(N) & Dmean(N) (left panels) and cell wall thickness CWTmax(N) & CWTmean(N) (right panels) for each individual tree. Markers of scatter plots (circles) represent actual tree rings; lines represent exponential functions of relationships. Data on maximum values of cell traits (Dmax, CWTmax) are presented in black color; data on mean values of cell traits (Dmean, CWTmean) are presented in grey color

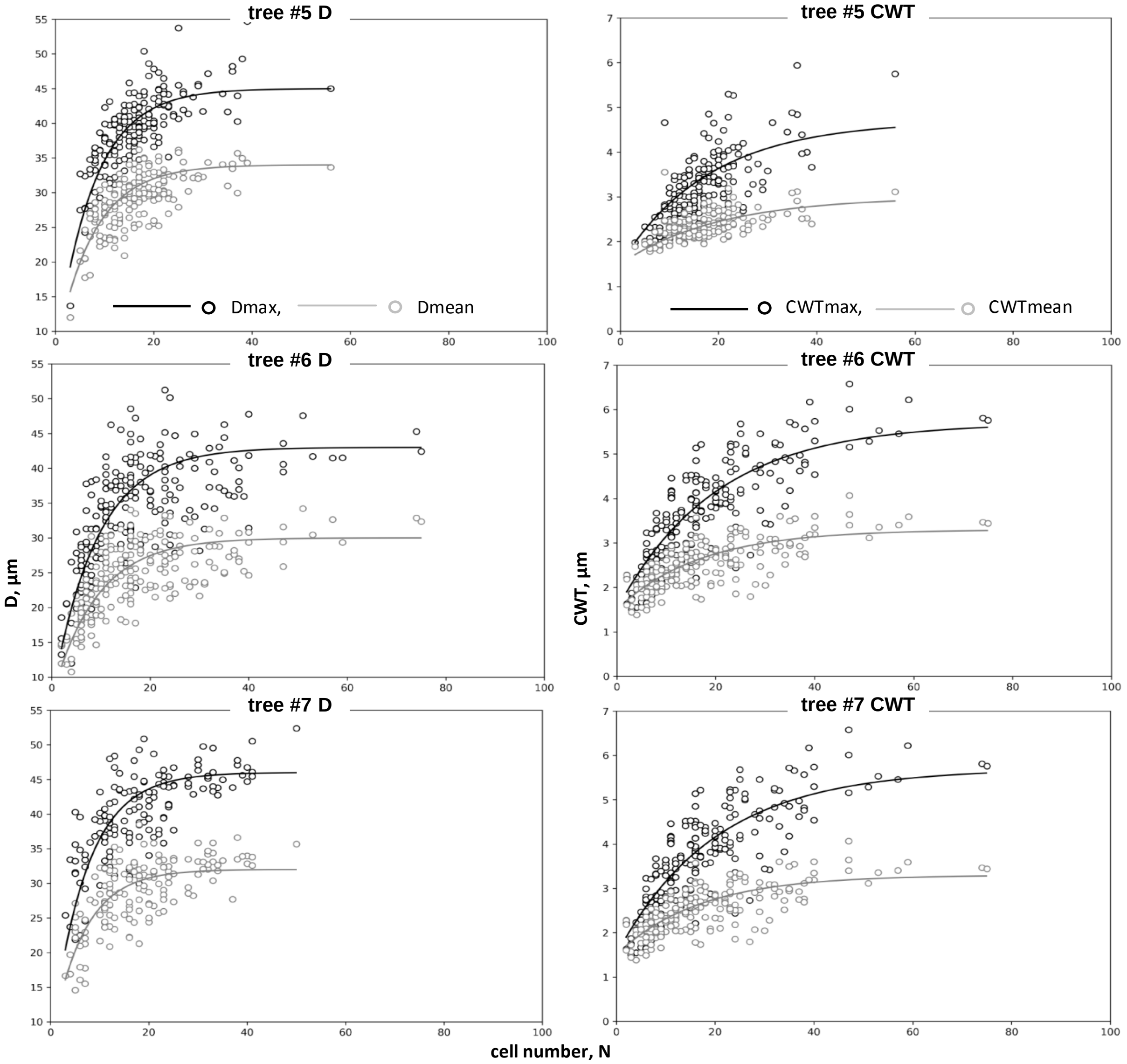


**Supplementary Figure S3.** Dependences of tracheid traits on cell number N for maximum & mean values of radial diameter Dmax(N) & Dmean(N) (left panels) and cell wall thickness CWTmax(N) & CWTmean(N) (right panels) for each individual tree. Continued

Supplementary Material

Dmax

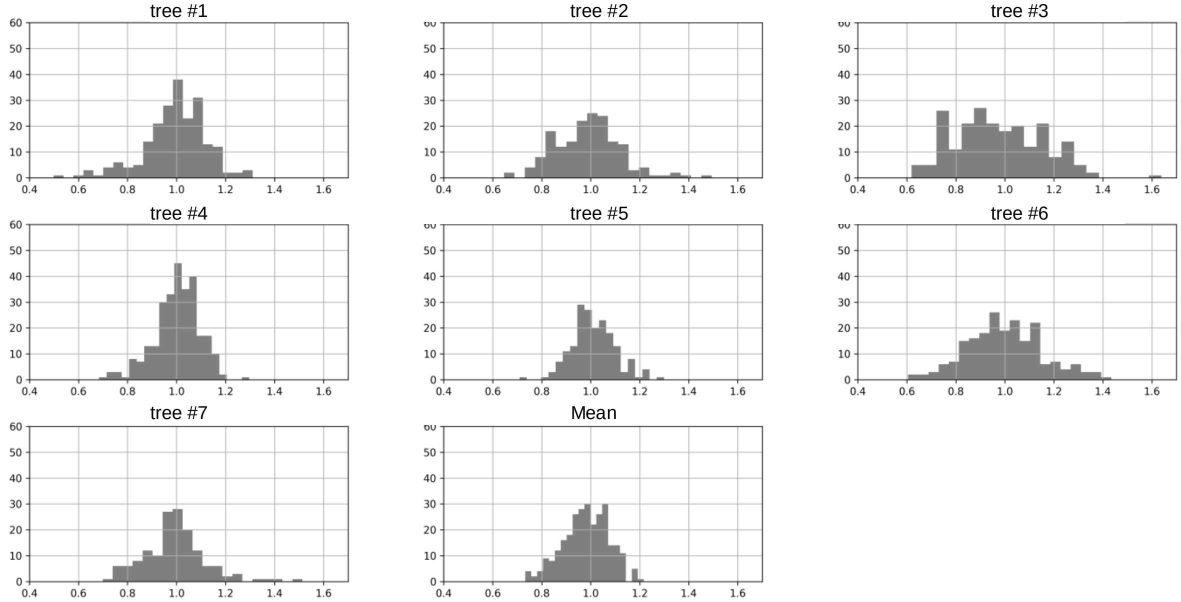


Dmean

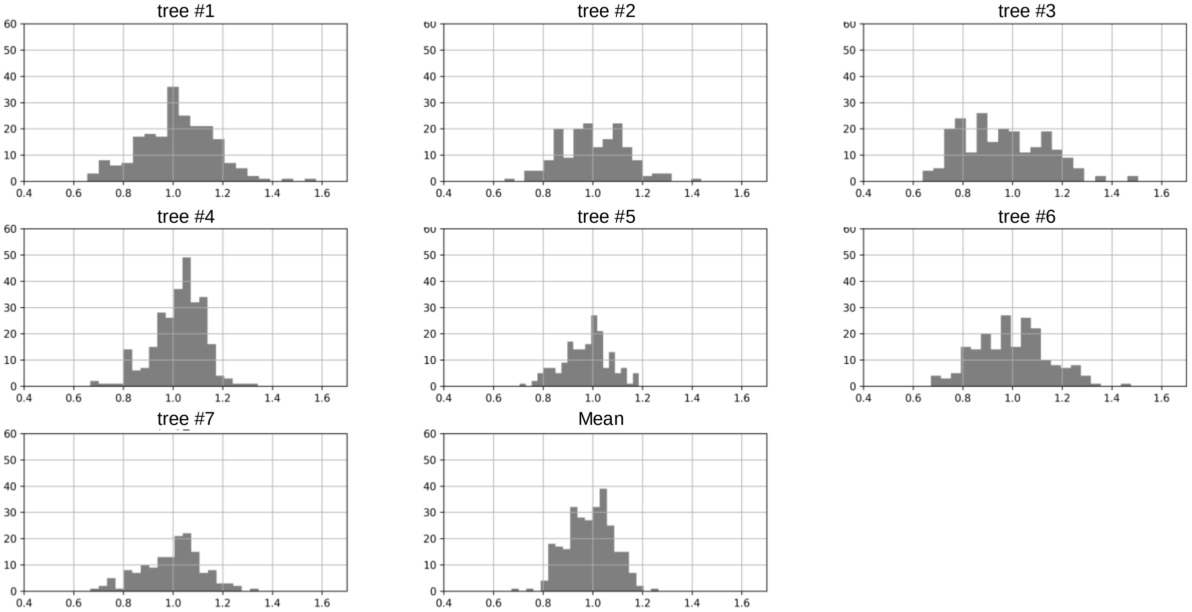


**Supplementary Figure S4.** Histograms of distribution frequencies for indexed anatomical characteristics of individual trees #1-7 and their averaged local chronologies (Mean)

CWTmax

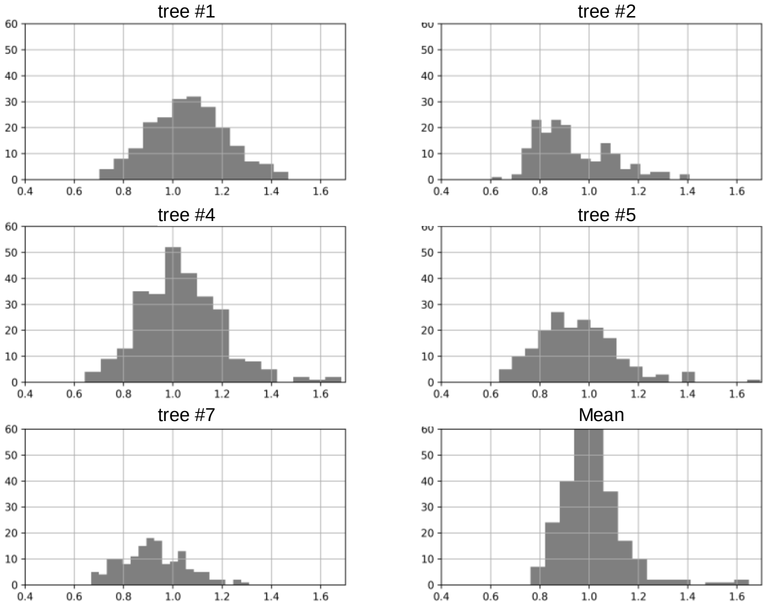


CWTmean

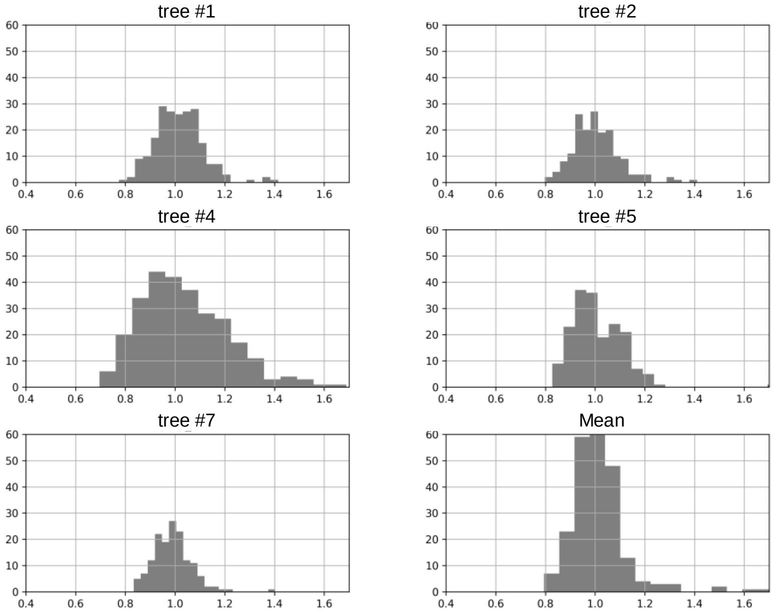


**Supplementary Figure S4.** Histograms of distribution frequencies for indexed anatomical characteristics of individual trees #1-7 and their averaged local chronologies (Mean). Continued

**Supplementary Table S1.** Statistics of raw TRW series of individual trees

| Tree | Min | Max | Mean | First Quartile | Third Quartile | Median | Standard deviation | Skewness | Kurtosis |
|---|---|---|---|---|---|---|---|---|---|
| **TRW** | | | | | | | | | |
| #1 | 0.03 | 2.04 | 0.61 | 0.31 | 0.83 | 0.50 | 0.41 | 1.18 | 1.17 |
| #2 | 0.06 | 2.31 | 0.63 | 0.37 | 0.79 | 0.55 | 0.39 | 1.59 | 3.34 |
| #3 | 0.02 | 3.23 | 0.83 | 0.39 | 1.15 | 0.65 | 0.63 | 1.28 | 1.48 |
| #4 | 0.06 | 1.23 | 0.33 | 0.21 | 0.39 | 0.30 | 0.18 | 1.51 | 3.26 |
| #5 | 0.04 | 1.90 | 0.50 | 0.31 | 0.62 | 0.47 | 0.27 | 1.43 | 2.96 |
| #6 | 0.02 | 2.44 | 0.48 | 0.20 | 0.64 | 0.39 | 0.40 | 1.94 | 5.48 |
| #7 | 0.05 | 1.79 | 0.56 | 0.31 | 0.76 | 0.49 | 0.35 | 0.82 | 0.31 |

**Supplementary Table S2.** Statistics of indexed anatomical characteristics of individual trees and their averaged local chronologies

| Tree | Min | Max | Mean | First Quartile | Third Quartile | Median | Standard deviation | Skewness | Kurtosis |
|---|---|---|---|---|---|---|---|---|---|
| **Dmax** | | | | | | | | | |
| tree #1 | 0.50 | 1.31 | 0.99 | 0.94 | 1.08 | 1.01 | 0.13 | -0.54 | 0.25 |
| tree #2 | 0.65 | 1.49 | 0.99 | 0.90 | 1.07 | 0.99 | 0.14 | 0.29 | -0.57 |
| tree #3 | 0.62 | 1.64 | 0.97 | 0.84 | 1.11 | 0.95 | 0.18 | 0.27 | -0.87 |
| tree #4 | 0.68 | 1.30 | 1.00 | 0.96 | 1.06 | 1.01 | 0.09 | -0.55 | 0.83 |
| tree #5 | 0.71 | 1.30 | 1.01 | 0.95 | 1.07 | 1.00 | 0.09 | 0.15 | -0.65 |
| tree #6 | 0.61 | 1.43 | 1.00 | 0.90 | 1.11 | 0.99 | 0.15 | 0.16 | -0.81 |
| tree #7 | 0.70 | 1.51 | 1.00 | 0.93 | 1.05 | 0.99 | 0.13 | 0.40 | -0.33 |
| Mean | 0.73 | 1.22 | 0.98 | 0.92 | 1.05 | 0.99 | 0.09 | -0.22 | -0.19 |
| **Dmean** | | | | | | | | | |
| tree #1 | 0.65 | 1.58 | 1.02 | 0.92 | 1.12 | 1.01 | 0.15 | 0.08 | -0.43 |
| tree #2 | 0.64 | 1.44 | 1.00 | 0.90 | 1.09 | 0.99 | 0.13 | 0.09 | -1.21 |
| tree #3 | 0.64 | 1.51 | 0.96 | 0.82 | 1.08 | 0.95 | 0.17 | 0.33 | -0.95 |
| tree #4 | 0.67 | 1.34 | 1.02 | 0.96 | 1.09 | 1.04 | 0.10 | -0.59 | 0.80 |
| tree #5 | 0.71 | 1.19 | 0.97 | 0.91 | 1.03 | 0.98 | 0.09 | -0.12 | -1.25 |
| tree #6 | 0.67 | 1.48 | 1.00 | 0.90 | 1.10 | 0.99 | 0.14 | 0.16 | -0.95 |
| tree #7 | 0.67 | 1.34 | 1.00 | 0.92 | 1.08 | 1.02 | 0.12 | -0.14 | -1.36 |
| Mean | 0.67 | 1.26 | 0.99 | 0.92 | 1.05 | 0.99 | 0.09 | -0.11 | -0.29 |
| **CWTmax** | | | | | | | | | |
| tree #1 | 0.70 | 1.88 | 1.07 | 0.96 | 1.16 | 1.06 | 0.17 | 0.57 | 0.97 |
| tree #2 | 0.61 | 1.41 | 0.94 | 0.81 | 1.05 | 0.90 | 0.16 | 0.45 | -1.20 |
| tree #3 | 0.65 | 1.35 | 0.97 | 0.87 | 1.08 | 0.97 | 0.15 | 0.10 | -1.13 |
| tree #4 | 0.64 | 1.94 | 1.04 | 0.93 | 1.13 | 1.03 | 0.18 | 1.11 | 3.51 |
| tree #5 | 0.63 | 1.69 | 0.95 | 0.84 | 1.05 | 0.93 | 0.16 | 0.55 | 0.14 |
| tree #6 | 0.55 | 1.36 | 0.99 | 0.90 | 1.10 | 0.99 | 0.16 | -0.15 | -0.94 |
| tree #7 | 0.67 | 1.31 | 0.92 | 0.84 | 1.01 | 0.91 | 0.13 | 0.19 | -1.48 |
| Mean | 0.76 | 1.94 | 1.02 | 0.94 | 1.07 | 1.00 | 0.14 | 2.61 | 11.78 |
| **CWTmean** | | | | | | | | | |
| tree #1 | 0.77 | 1.42 | 1.02 | 0.95 | 1.08 | 1.02 | 0.10 | 0.52 | 0.55 |
| tree #2 | 0.80 | 1.41 | 1.01 | 0.94 | 1.05 | 1.00 | 0.10 | 0.61 | 0.08 |
| tree #3 | 0.69 | 1.35 | 0.98 | 0.89 | 1.05 | 0.98 | 0.12 | 0.19 | -0.83 |
| tree #4 | 0.70 | 2.02 | 1.05 | 0.91 | 1.15 | 1.02 | 0.19 | 1.14 | 2.72 |
| tree #5 | 0.83 | 1.74 | 1.01 | 0.94 | 1.08 | 0.99 | 0.11 | 1.21 | 5.37 |
| tree #6 | 0.61 | 1.37 | 0.99 | 0.90 | 1.07 | 1.00 | 0.13 | -0.15 | -0.76 |
| tree #7 | 0.83 | 1.40 | 0.99 | 0.94 | 1.03 | 0.99 | 0.08 | 0.62 | 0.94 |
| Mean | 0.79 | 2.02 | 1.02 | 0.96 | 1.05 | 1.00 | 0.13 | 3.70 | 20.92 |

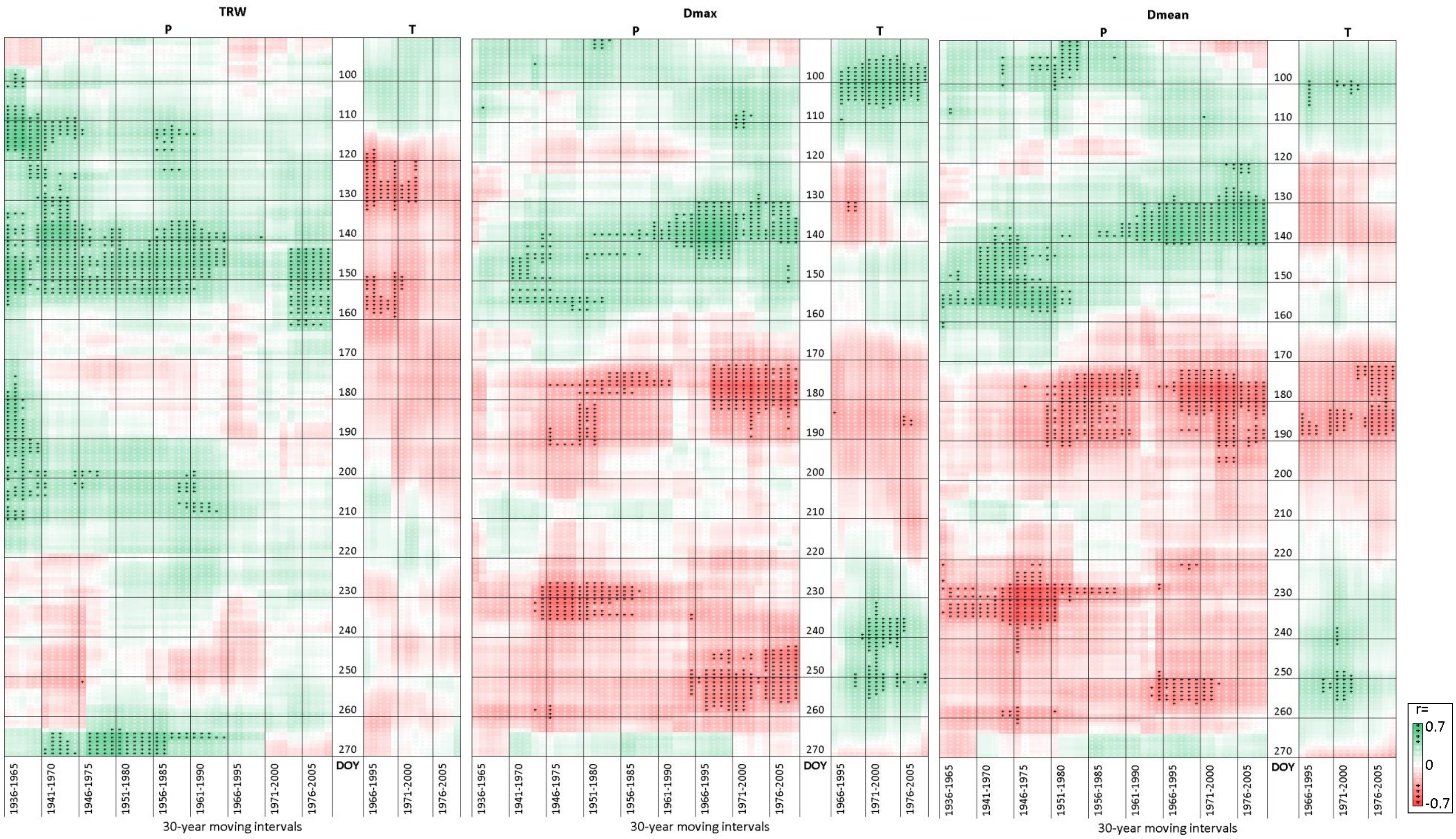


**Supplementary Figure S5.** Temporal stability of pine climatic response: moving 30-year correlation coefficients of indexed TRW and anatomical chronologies with the same 21-day series of precipitation and temperature as in Fig. 6. Correlations are centered within season, i.e. points at DOY 90 represent series from DOY 80 to DOY 100, etc. Values of correlation coefficients are coded with color gradient, significant correlations at $p<0.05$ are marked with black dots (see legend)

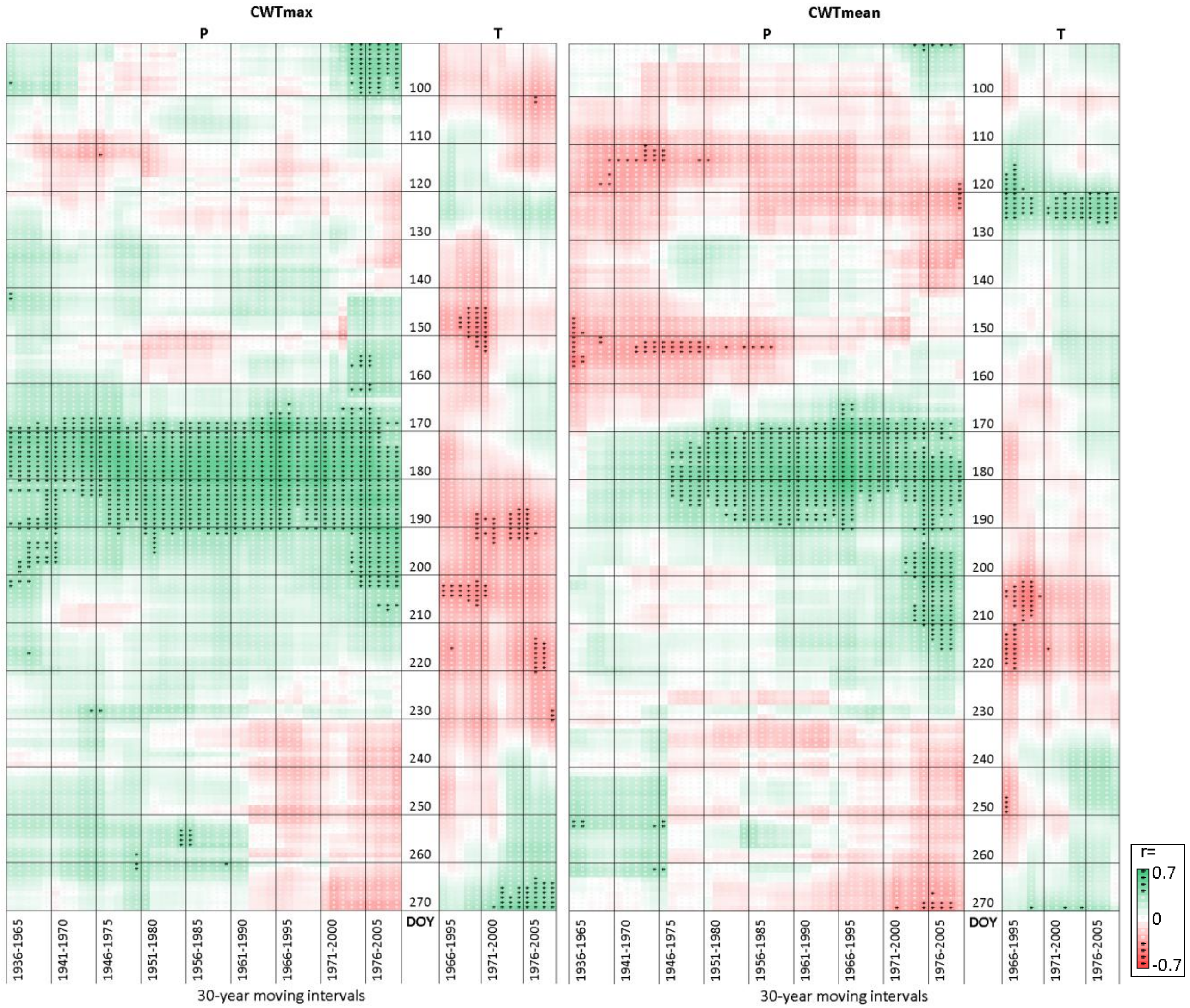


**Supplementary Figure S5.** Temporal stability of pine climatic response. Continued
